# How supermarket retailers value business outcomes of healthy food retail strategies: a discrete choice experiment

**DOI:** 10.3389/fpubh.2024.1450080

**Published:** 2024-11-08

**Authors:** Moosa Alsubhi, Miranda R. Blake, Ann Livingstone, Marj Moodie, Jaithri Ananthapavan

**Affiliations:** ^1^Deakin Health Economics, Institute for Health Transformation, School of Health and Social Development, Faculty of Health, Deakin University, Geelong, VIC, Australia; ^2^Global Centre for Preventive Health and Nutrition, Institute for Health Transformation, School of Health and Social Development, Faculty of Health, Deakin University, Geelong, VIC, Australia

**Keywords:** healthy food retail, business outcomes, supermarkets, grocery stores, discrete choice experiment, willingness to pay

## Abstract

**Background:**

Supermarkets are businesses, and any voluntary changes to increase the healthiness of their food offerings must align with retailers’ commercial needs. Business outcomes of healthy food retail strategies are important non-health factors that may influence retailers’ decisions to implement these strategies. Although there is growing evidence on the significance of various business outcomes, such as net profit and customer satisfaction, it remains unclear how retailers value and trade-off these outcomes against each other. This study aimed to determine retailer preferences and measure their marginal willingness to pay for key business outcomes.

**Methods:**

A Discrete Choice Experiment (DCE) survey recruited current or former owners or managers of supermarkets or grocery stores in Australia. It included 12 choice tasks for two hypothetical scenarios (A or B) that the retailer could implement in their store, along with an option to maintain the current situation (opt-out option). The survey included six attributes (net profit, healthy items sold, customer and retailer satisfaction, ease and costs of implementation) with 3–4 levels each. A multinomial logit model was used to estimate preferences and calculate marginal rates of substitution and marginal willingness to pay.

**Results:**

Sixty-one respondents completed the DCE, resulting in a 72% response rate. Retailers identified customer satisfaction as the highest ranked business outcome when deciding to implement healthy food retail strategies. This was followed by the percentage of healthy items sold, supplier satisfaction, net profit, implementation cost, and ease of implementation. The marginal willingness to pay for different attribute levels varied from A$650 per year per store for a strategy that increases net profit by 3% to A$32,136 for a strategy leading to “very satisfied” levels of customer satisfaction compared to the base level.

**Conclusion:**

The results could be used to guide the implementation of healthy food retail strategies that also meet the needs of retailers.

## 1 Background

What people eat is an important determinant of their health. Supermarkets and grocery stores are the key settings for purchasing food and drink in many countries including Australia (1, 2). Approximately 67% of Australians’ purchasing of food and drink takes place in supermarkets and grocery stores (3). Supermarkets are businesses, so any voluntary changes to increase the healthiness of their food offerings also need to meet retailers’ commercial needs (4). Healthy food retail strategies are business practices or operations that promote healthy food and/or restrict unhealthy food within supermarkets to encourage the purchase and consumption of healthier foods. These may include pricing strategies such as discounts on healthy food, placement or positioning of healthy food at prime locations such as near checkouts, increased availability of healthy alternatives, and promotion of healthy products within stores using advertising, nutritional labelling and signage. Business outcomes of healthy food retail strategies are key non-health or non-nutritional outcomes such as net profit, percentage of healthy items sold, customer satisfaction that may influence retailer decision-making related to the implementation and maintenance of healthy food retail strategies (4). The success of healthy food retail strategies such as choice architecture and marketing strategies (altering product placement, product characteristics, price, and promotion) to improve nutrition outcomes may be affected by the impact of these strategies on retailer business outcomes.

Including retailer preferences and understanding the relative importance of different business outcomes may contribute to the success of healthy food retail strategies by identifying strategies where there is synergy between health and business outcomes (5–7). There are two main categories of valuation techniques that have been used to estimate preferences. In revealed preference studies, people’s preferences are observed in the actual market situation, whereas stated preference methods elicit preferences using surveys to present hypothetical situations. There are different types of stated preference elicitation techniques including standard gamble, time trade-off, person trade-off, contingent valuation (CV), and discrete choice experiment (DCE) (8). The two most common methods are DCE and CV (9).

In health economics, DCEs are commonly used to elicit preferences and value for aspects of healthcare products and interventions that go beyond health outcomes. DCEs have been used in health economics to elicit stakeholders’ preferences for screening programs (10, 11), prevention programs (12), treatment (13), rehabilitation (14), access to services (15), and health insurance (16). While DCEs have been used to elicit stakeholders’ preferences to support the prioritisation, design, and implementation of healthcare interventions (17, 18), there has been limited application of DCEs to health promotion strategies targeting food retail in supermarkets and grocery stores.

To our knowledge, no previous study has used a DCE or any other preference elicitation technique to assess how retailers quantify and trade-off business outcomes of healthy food retail strategies. A systematic review of healthy food retail economic evaluations showed that only limited perspectives have been used in economic evaluations of healthy food retail strategies, and none have incorporated retailer perspectives and valuations (19). This study will enable us to understand how retailers value business outcomes resulting from healthy food retail strategies and quantify preferences informing future economic evaluations using a broader societal perspective. For example, the evaluation of a healthy food retail intervention may include an assessment of consumer satisfaction with the intervention. The change in consumer satisfaction can then be monetised using the marginal willingness to pay (mWTP) from this DCE to produce a more comprehensive cost–benefit analysis of the intervention. This study aimed to (i) elicit retailer preferences of key business outcomes, and (ii) quantify mWTP for retailer business outcomes.

## 2 Methods

### 2.1 Study design

DCE is a quantitative technique used to elicit people’s preferences without asking them directly to state their preferred options. In a DCE, respondents are asked to trade-off between two or more hypothetical scenarios. Each scenario has several attributes, and each attribute has several levels. Across all choice tasks, attributes remain the same whilst levels are varied to elicit preferences and trade-offs between different attributes and levels. Attributes refer to variables that represent the most important characteristics of the situation or scenario. DCEs have the advantages of providing quantitative information on the strength of preference, quantifying and measuring monetary and non-monetary values or outcomes for inclusion in economic evaluation and decision-making, and allowing investigation of various types of questions (20, 21). A DCE was chosen in the current study because it more accurately reflects the real-word decision-making context compared to some other stated preference methods (22). This study was undertaken in accordance with the ESTIMATE checklist for DCEs (Supplementary file 1) (23). Ethics approval for this study was obtained from the Faculty of Health Human Ethics Advisory Group (HEAG) at Deakin University (HEAG-H 156_2022).

### 2.2 Participants

Self-identified owners and managers of supermarkets and grocery stores in Australia were recruited as participants. Our recruitment target for this study was 60 respondents. There is no agreement among researchers about the appropriate sample size for a DCE. Lancsar and Louviere (20) suggested that 20 observations per choice set is suitable to achieve reliable results (20, 21). The sample size was determined based on estimates from Ngene (a software used to design DCEs). The Ngene design generated an ‘S estimate’ (or sample size estimate of 8.6,D-error = 0.01014), meaning the design was robust with only nine participants. We engaged with an external panel provider, Qualtrics, to recruit participants. Web panels are widely used to recruit participants for survey studies (24). Recent DCE studies in the Australian context used online platforms panel to recruit participants (25–27). Qualtrics only distributed the survey to panel members who were likely to meet the inclusion criteria. Before we engaged with Qualtrics to recruit participants through their panel, we tried to recruit participants through different channels including University and project websites, and X and LinkedIn social media accounts, but only 11 participants completed the survey. This led us to recruit additional participants through Qualtrics.

To be included in this study, participants needed to self-identify as current or previous owners or managers of supermarkets or grocery stores in Australia. The opinions of chain supermarket managers may be affected by the corporate strategy and culture of the organization. While the final decision around policy adoption are likely to take place at the chain headquarters, chain store managers are responsible for the implementation and follow-up of different promotion strategies in their stores (including healthy food strategies). They are aware of the various business outcomes that affect the implementation of healthy food retail strategies such as customer satisfaction, ease of implementation, retailer and supplier satisfaction, and commercial viability (4, 28). The survey was piloted with grocery store owners and chain supermarket managers to make sure they understood various business outcomes included in the survey and also to check if they could make informed choices regarding the importance of varied business outcomes relevant to this DCE. Employees within supermarkets and grocery stores were excluded due to their lack of authority to make decisions around the implementation of healthy food retail strategies. Owners or managers of restaurants or other facilities where people eat prepared meals on-site such as cafeterias and canteens in streets, supermarkets, schools, and workplaces were excluded.

### 2.3 Development of attributes and levels

A three-stage iterative process was used to develop the attributes, namely (i) a literature review of existing business outcomes, (ii) prioritisation (ranking) cross sectional-survey with retailers, and (iii) discussion among the research team to select the final attributes. A literature search found 10 reviews which identified 87 relevant business outcomes (29). Attributes with similar meanings were combined and then prioritised based on the frequency of being reported in the literature. Of the 87 business outcomes identified in the literature, 17 were included in the survey. The survey was piloted with retailers (three grocery store owners and three chain supermarket managers) and with academics who work with retailers (10 academic experts from the National Health and Medical Research Council (NHMRC) Centre of Research Excellence in Food Retail Environments for Health (30)). At this stage we asked retailers and experts to comment on the language, clarity, and flow of the survey, and to identify any additional business outcomes that should be included in the list. No additional outcomes were identified.

The survey was completed by 97 grocery store owners and supermarket managers (unpublished results) to ascertain the most important business outcomes for retailers when making decisions to implement healthy food retail strategies. Participants were asked to rank these outcomes from 1 representing the most important outcome, followed by 2, and so on across four domains (commercial viability, retailer perception, consumer perception, community outcomes) (4). The top two most important business outcomes within each domain were selected which included: (i) competitiveness of store offers for healthier products compared to other competitors in the nearby area, (ii) store foot traffic and customer loyalty to the store, (iii) the satisfaction of stakeholders including food suppliers and producers with the intervention promoting healthier products, (iv) ease of implementation for employees, (v) customer satisfaction with the strategy, (vi) customer demand for new or existing healthy food and drinks at your store, (vii) the proportion of healthy promoted food and drinks items sold and (viii) the net profit generated by selling healthy promoted products. Finally, these findings were discussed with the research team to finalise attributes for the current DCE study. Decisions focused on ensuring that there wasn’t any overlap between attributes and the ease of measuring and trading off the attributes in a DCE task. “Customer loyalty to the store” and “competitiveness of the store offers” were excluded by the research team because of the difficulty of quantifying these two attributes. Also, “customer demand for healthy food and drinks” and “proportion of healthy promoted food and drinks items sold” were combined because they represented the same concept. The cost attribute was included to estimate retailers’ willingness to pay for the characteristics of their preferred strategy. Six attributes were included in the final DCE survey: (i) change in percentage of the store’s net profit; (ii) change in percentage of healthy items sold; (iii) ease of implementing and maintaining the strategy for retail staff; (iv) satisfaction of food suppliers with the strategy; (v) customer satisfaction with the store overall as a result of the strategy; and (vi) costs associated with implementing and maintaining the strategy in each store per year.

Discussion among the research team supported by the literature review were used to assign levels for each attribute. The change in percentage of the store’s net profit was informed by grey literature searching (e.g., government reports, industry association websites, etc.), which indicated that the net profit generated by supermarket and grocery stores ranged from approximately 1 to 5% (31, 32). The percentage change in healthy items sold was informed by a meta-analysis which demonstrated that strategies (e.g., place, profile, portion, pricing, promotion, healthy default picks, prompting, and proximity) overall had a small significant effect size (Cohen’s d on food purchase behaviour, d = 0.17, 95% CI [0.04, 0.09]) (33). The estimated costs of implementing healthy food retail strategies reported in the academic literature (e.g., shelf labelling, promotion, and calorie labelling) ranged from A$1,545 to A$3,700 (mean: A$2,527) per store per year (34, 35). The range for the cost attribute levels should incorporate values that are higher than the market price, because this may not be the maximum amount that respondents are willing to pay (36); therefore the maximum cost attribute was set at A$10,000 per store per year. Three-point Likert scales using star symbols were used to quantify supplier and customer satisfaction (1 star = very unsatisfied, 3 stars = neutral, 5 stars = very satisfied). The following categorical levels were used to value the ease of implementation attribute: ‘easy’, ‘neither easy nor difficult’, and ‘difficult’. The final attributes and levels are presented in Table 1.

**Table 1:** Final discrete choice experiment attributes and levels.

No	Attribute	Levels	Levels type	Coding
1	The change in the store’s net profit	0% increase in store’s net profit	Continuous	0 (reference)
		3% increase in store’s net profit		3
		6% increase in store’s net profit		6
2	The change in percentage (%) of healthy items sold	0% increase in healthy items sold	Continuous	0 (reference)
		3% increase in healthy items sold		3
		6% increase in healthy items sold		6
3	Ease of putting in place and maintaining the strategy for retail staff	Difficult	Categorical	0 (reference)
		Neither easy nor difficult		1
		Easy		2
4	Satisfaction of food suppliers with the strategy	★✰✰✰✰ (very unsatisfied)	Categorical	0 (reference)
		★★★✰✰ (neutral)		1
		★★★★★ (very satisfied)		2
5	Customer satisfaction with store overall as a result of the strategy	★✰✰✰✰ (very unsatisfied)	Categorical	0 (reference)
		★★★✰✰ (neutral)		1
		★★★★★ (very satisfied)		2
6	Costs associated with putting in place and maintaining the strategy in each store per year	A$1,000	Continuous	1,000 (reference)
		A$4,000		4,000
		A$7,000		7,000
		A$10,000		10,000

### 2.4 Experimental design

An initial D-efficient design was constructed using Ngene assuming zero prior coefficients (37). The survey was piloted with six respondents who represented 10% of the final sample size. A multinominal logit (MNL) model was used to analyse data and identify priors using STATA/IC 16.1 (38). Prior coefficients derived from the pilot study were used to construct the final unlabelled design (D-error = 0.01014). See Supplementary file 2 for final D-efficient design. The final design included three unlabelled alternatives (Option A, Option B or Option C (opt-out option)) and six attributes (five with 3 levels and one with 4 levels). Inclusion of the opt-out option reflected the real-world scenario where respondents may decide not to implement any healthy food retail changes. Also, forcing respondents to make a choice can lead to overestimation of the utility of the parameters (9). The final design consisted of 12 choice tasks per respondent. Respondents were asked to consider each choice task as if they were deciding which health promotion strategy option (A or B) they would use in their store. Respondents could then state that they preferred to remain in the current situation (not to use any healthy promoting strategies) by choosing “opt-out option” (Option C).

### 2.5 Piloting and online procedures

The survey (Supplementary file 3) was built in the Qualtrics online survey platform and pre-tested with 10 academic experts who work with supermarket/grocery retailers and then pilot tested with six supermarket/grocery store owners and managers. Pilot responses were not included in the final dataset. Minor changes to wording were made to the survey following the piloting. The survey was self-administered using Qualtrics, over a two-week period in July 2023. The survey started with a definition of healthy food retail strategies and examples to ensure participants understood the choice context and screening question. This was followed by 12 choice tasks, and a final section on respondent characteristics (e.g., type of store respondents has managed/owned, location of stores, years of experience in the grocery industry). The survey was designed to be completed in 10 min or less. Responses of respondents who did not give consent or did not complete the survey were not included in the final analysis. Also, respondents who selected the same option for every task were excluded. On completion, respondents received a small compensation for their time from Qualtrics as part of their usual rewards program, such as e-gift vouchers, and flight points. Giving incentives to participants has been found to increase response rate and to not affect the quality of the survey results (39).

### 2.6 Statistical analysis

Data collected from all respondents were entered, coded, and cleaned using Microsoft Excel and then transferred into STATA/IC 16.1 for analysis. In this study, three attributes (percentage of items sold, percentage of net profit, costs of implementation) were continuous variables, whereas customer satisfaction, supplier/producer satisfaction, and ease of implementation were dummy-coded (Table 1). Respondent characteristics (e.g., position, store location, experience, etc.) were summarised descriptively. A conditional logit (CL) model was used to analyse the DCE data. Such a multinomial logit model (MNL) is widely used to analyse DCE data which relies on the mean preference of the entire sample (23). In this study, the choice between alternatives was modelled based on the characteristics of the alternatives rather than the characteristics of the respondents making the choices, therefore using MNL suited the primary aim of this DCE (40). First, a linear CL model was used to assess the theoretical validity of the attributes’ coefficients. Then, the data obtained from all respondents was analysed to elicit the relative importance of attributes and attribute levels. The relative importance of each attribute was calculated as the (absolute) difference in preference weight (utility) between the highest and lowest (reference) levels (41). In addition, the mWTP estimates for each attribute were calculated by dividing the coefficient of each attribute by minus one times the coefficient of the linear term of costs expressed in Australian dollars (42, 43). The estimates represent the mWTP of the average respondent to switch from the base (reference) level to another level. A positive coefficient indicates retailers are willing to pay more for the attribute, whereas a negative coefficient suggests that retailers would want compensation in order to accept the attribute. Subgroup analyses by type of stores (supermarkets versus grocery stores) were performed. Respondents who had worked across both chain supermarkets and independent grocery stores were grouped based on their current position. A stability validity test was used to assess the internal validity by repeating the same choice task twice. Throughout the analysis, the level of significance was set at *p* < 0.05.

## 3 Results

### 3.1 Respondents’ characteristics

Of the 105 respondents who clicked the online survey link, 76 proceeded to complete the survey (response rate = 72%). Ten responses were excluded because respondents did not identify themselves as an owner or manager of Australian supermarkets or grocery stores. Another five responses were excluded because respondents consistently selected the same option for every task. Most responses were completed in less than 1 min; suggesting that participants did not take enough time to read the questions and make informed decisions. Therefore, these five responses were excluded from the analysis. Sixty-one responses (2,190 observations) were included in the final analysis. Seventy-three observations (3.3%) were counted for the opt-out scenario. The mean time to complete the survey was approximately 7 min. The results of the stability validity test suggested that 82% of respondents selected the same alternative in both identical choice tasks. Most respondents self-identified as managers (75%) and worked in chain supermarkets (70%). This is consistent with the current market share of chain and independent supermarkets/ grocery stores in Australia (44). The majority of respondents work or have worked in NSW (38%) or Victoria (29%; the two most populous states in Australia). Sixty-one percent of respondents had between 1 and 5 years of experience in their position. Respondent characteristics are presented in Table 2.

**Table 2:** Characteristics of respondents.

Measure	Items	Frequency	Percentage
Position	Manager	42	69%
	Owner	19	31%
Location of participants’ store	Australian Capital Territory	0	0%
	New South Wales	23	38%
	Northern Territory	1	2%
	Queensland	9	15%
	South Australia	5	8%
	Tasmania	1	2%
	Victoria	18	29%
	Western Australia	4	6%
Type of stores participants have worked in/owned	Chain supermarket	43	70%
	Independent grocery store	11	18%
	Both	7	12%
Experience in grocery industry	Less than 1 year	7	12%
	Between 1 and 5 years	38	62%
	Between 6 and 10 years	10	16%
	More than 10 years	6	10%

### 3.2 Results of the linear conditional logit model

Table 3 presents the results of the linear CL model. Across the total sample, four of the six attributes had positive coefficients, all of which were statistically significant (*p* < 0.05) except for the attribute ‘ease of implementation’ (*p* = 0.311). The cost coefficient had a negative effect on retailers’ decision-making to implement healthy strategies. The regression coefficients across the total sample displayed the expected signs, which confirmed the theoretical validity of our estimates. A similar pattern was observed for both the independent grocery stores (Supplementary file 4) and chain supermarkets (Supplementary file 5). However, in both subgroup analyses, only supplier and customer satisfaction were statistically significant. No significant changes were observed in the results of linear CL model across all the sample after excluding those who failed the validity test (Supplementary file 6).

**Table 3:** All respondent results of the linear conditional logit model (CL).

Attribute (business outcomes)	Coefficient	SE	[95% CI]	*p* value
Net profit	0.047	0.018	[0.012; 0.082]	0.009
Percentage (%) of healthy items sold	0.130	0.025	[0.080; 0.178]	< 0.001
Ease of implementation	0.071	0.070	[−0.067; 0.209]	0.311
Supplier/producer satisfaction	0.375	0.064	[0.250; 0.500]	< 0.001
Customer satisfaction	0.542	0.80	[0.384; 0.699]	< 0.001
Implementation costs	−0.0000303	0.0000167	[−0.0000631; 0.0000251]	0.070
Number of observations: 2,190. Wald chi^2^ (6): 93.62. Prob > chi^2^: *p* < 0.0001. Log pseudo likelihood: −604.523.				

CI, confidence interval; SE, standard error. Positive coefficients indicated that business outcomes had a positive impact on retailers’ preference to implement healthy food interventions in their store, while outcomes with negative coefficients had a negative effect on their preferences.

### 3.3 Relative importance of business outcomes

**Figure 1:**
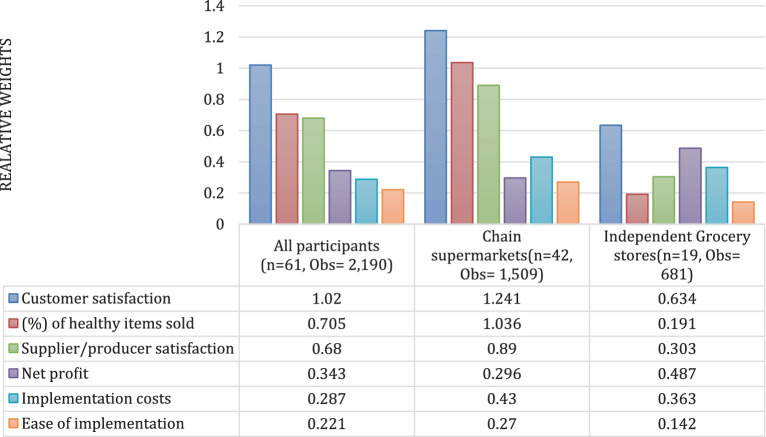
Relative importance of business outcomes for all respondents and by type of store. Model estimates of preferences for attribute-levels.

Figure 1 and Table 4 present the relative importance of various business outcomes using the CL model (main effects) across the total sample, chain supermarket respondents, and independent grocery store respondents. Results across the total sample indicated that customer satisfaction (relative weight difference between the highest and lowest levels = 1.020) was the most important business outcome for retailers in their decision-making to implement healthy food strategies, followed by percentage of healthy items sold (weighted difference = 0.705), supplier/producer satisfaction (weighted difference = 0.680), net profit (weighted difference = 0.343), implementation cost (weighted difference = 0.287) and ease of implementation (weighted difference = 0.221). Subgroup analyses showed that supplier and consumer satisfaction were the only attributes that were statistically significant across both groups. No significant changes were observed in the relative importance of business outcomes after excluding the participants who failed the validity test (Supplementary file 7).

**Table 4:** Model estimates of preferences for attribute-levels.

Attribute (business outcome)	Attribute level	All respondents (*n* = 61)		Chain supermarkets (*n* = 42)		Independent grocery stores (*n* = 19)	
		Coefficient (SE)	[95% CI]	Coefficient (SE)	[95% CI]	Coefficient (SE)	[95% CI]
Customer satisfaction	Very unsatisfied (reference)						
	Neutral	0.882 (0.310) **	[0.0275; 1.489]	1.142 (0.393) **	[0.371; 1.914]	0.429 (0.485)	[−0.521; 1.380]
	Very satisfied	1.020 (0.247) ***	[0.535; 1.504]	1.241 (0.325) ***	[0.603; 1.878]	0.634 (0.420)	[−0.189; 1.458]
Percentage (%) of healthy items sold	0% (reference)						
	3% increase	0.209 (0.277)	[−0.334; 0.752]	0.456 (0.350)	[−0.226; 1.138]	−0.165 (0.460)	[−1.068; 0.738]
	6% increase	0.705 (0.323) **	[0.072; 1.338]	1.036 (0.413) **	[0.227; 0.1845]	0.190 (0.525)	[−0.838; 1.219]
Supplier/producer satisfaction	Very unsatisfied (reference)						
	Neutral	0.442 (0.294)	[−0.134; 1.018]	0.586 (0.396)	[−0.190; 1.361]	0.117 (0.484)	[−0.832; 1.065]
	Very satisfied	0.680 (0.225) **	[0.240; 1.120]	0.890 (0.281) **	[0.340; 1.440]	0.303 (0.391)	[−0.464; 1.070]
Net profit	0% (reference)						
	3% increase	0.043 (0.216)	[−0.380; 0.466]	0.174 (0.304)	[−0.422; 0.770]	−0.167 (0.274)	[−0.704; 0.370]
	6% increase	0.343 (0.151) **	[0.046; 0.639]	0.296 (0.191)	[−0.079; 0.671]	0.487 (0.272)	[−0.047; 1.021]
Ease of implementation	Difficult (reference)						
	Neither easy nor difficult	0.135 (0.130)	[−0.121; 0.391]	0.126 (0.180)	[−0.136; 0.568]	0.045 (0.218)	[−0.383; 0.473]
	Easy	0.221 (0.157)	[−0.086; 0.528]	0.270 (0.217)	[−0.154; 0.695]	0.142 (0.213)	[−0.276; 0.559]
Implementation costs	A$1,000 (reference)						
	A$4,000	−0.200 (0.321)	[−0.830; 0.429]	−0.430 (0.421)	[−1.255; 0.394]	0.173 (0.494)	[−0.795; 1.14]
	A$7,000	−0.274 (0.331)	[−0.922; 0.374]	−0.343 (0.470)	[−1.266; 0.578]	−0.163 (0.447)	[−1.038; 0.713]
	A$10,000	−0.287 (0.192)	[−0.664; 0.089]	−0.227 (0.273)	[−0.762; 0.308]	−0.363 (0.293)	[−0.938; 0.211]
		Number of observations: 2,190 Wald chi^2^ (6): 105.12 Prob > chi^2^: *p* < 0.0001 Log pseudo likelihood: −603.343		Number of observations: 1,509 Wald chi^2^ (6): 111.16 Prob > chi^2^: *p* < 0.0001 Log pseudo likelihood: −382.649		Number of observations: 681 Wald chi^2^ (6): 35.11 Prob > chi^2^: 0.0008 Log pseudo likelihood: −211.109	

CI, confidence interval; SE, standard error. Statistical significance is indicated at <0.01 (***), <0.05 (**).

Table 4 shows the results of the CL model estimates of retailers’ preferences for specific attribute levels. For all respondents, preferences consistently increased with the addition of more benefits for the following attributes: customer satisfaction, percentage of healthy items sold, supplier and producer satisfaction, net profit, and ease of implementation. For example, retailers preferred a 6% increase in percentage of healthy items sold compared to a 3% increase and the base level (0% increase). Also, results showed that retailers expressed greater preference for customers/suppliers being “very satisfied,” compared to “neutral” or “very unsatisfied.” Costs were associated with negative coefficients, which implied that as the cost of intervention increases, preference for the implementation of healthy food retail strategies in supermarkets and grocery stores decreased. Similar patterns were observed among chain supermarket respondents. However, the results for the independent grocery store respondents were not consistent with the chain supermarket subgroup and the whole sample; this may be due to the relatively small (statistically insignificant) sample size. For example, our results showed a difference between chain supermarket managers (coefficient = +0.174) and small grocery store owners (coefficient = −0.167) when responding to a 3% net profit change as shown in Table 4.

### 3.4 Marginal willingness to pay (mWTP) estimates

Table 5 presents retailers’ mWTP to switch from the base (reference) level to another level for various attributes across the total sample. The magnitude of mWTP for various attribute levels varied from A$650 per year for a strategy that increased the net profit by 3% (statistically non-significant) to A$32,136 per year for a strategy that led to “very satisfied” levels of customer satisfaction. Compared to the reference level (very unsatisfied), retailers were willing to pay A$27,399 and A$32,136 per year for healthy food interventions that had neutral customer satisfaction and high customer satisfaction, respectively. Also, results showed that retailers were willing to pay A$22,446 per year for a healthy strategy that increased the percentage of healthy items sold by 6% from the reference level (0%). Similar patterns were observed across other attributes (supplier satisfaction, net profit, ease of implementation). Subgroup analyses by type of store (chain supermarkets and independent grocery stores) are presented in Supplementary files 8, 9. The mWTP for all attributes across the independent grocery stores ranged from −A$2,448 (statistically non-significant) to A$12,402. The mWTP for all attributes across the chain supermarkets respondents ranged from A$4,744 to A$57,222.

**Table 5:** Marginal willingness to pay for attribute levels for all respondents.

Attribute (business outcome)	Attribute level	Coefficient (SE)	mWTP [A$, 95% CI]
Customer satisfaction	Very unsatisfied (reference)		
	Neutral	0.885 (0.321) ***	27,399 [7,920; 46,877]
	Very satisfied	1.038 (0.182) ***	32,136 [21,091; 42,180]
Percentage of healthy items sold	0% (reference)		
	3% increase	0.257 (0.274)	7,957 [−8,670; 24,584]
	6% increase	0.725 (0.198) ***	22,446 [10,432; 34,461]
Supplier/producer satisfaction	Very unsatisfied (reference)		
	Neutral	0.467 (0.292)	14,458 [−3,260; 32,176]
	Very satisfied	0.667 (0.160) ***	20,650 [10,940; 30,359]
Net profit	0% (reference)		
	3% increase	0.021 (0.210)	650 [−12,094; 13,394]
	6% increase	0.327 (0.146) **	10,124 [1,265; 18,983]
Ease of implementation	Difficult (reference)		
	Neither easy nor difficult	0.133 (0.132)	4,118 [−3,893; 12,129]
	Easy	0.216 (0.156)	6,687 [−2,780; 16,154]
Implementation costs (Per year)	Linear	−0.0000323 (0.0000184)	-

CI, confidence interval; mWTP, marginal willingness to pay; SE: standard error. Statistical significance is indicated at <0.01 (***), <0.05 (**).

## 4 Discussion

This is the first study that has used a DCE to assess how retailers quantify and trade-off business outcomes when deciding whether to implement healthy food retail strategies in supermarkets and grocery stores The results of this study indicated that customer satisfaction was the most important business outcome for supermarkets and grocery store retailers in their decision-making to implement healthy food strategies, followed by percentage of healthy items sold, supplier/producer satisfaction, net profit, implementation cost, and ease of implementation.

Previous quantitative and qualitative studies have indicated the importance of these business outcomes in retailer decision-making to implement healthy food retail strategies (45–48). Three studies have shown that customer satisfaction and demand for healthier products are the top priorities in retailer decision-making. Some studies have demonstrated the importance of supplier satisfaction in retailers’ decisions to implement healthy strategies in their store. For example, Houghtaling et al. (45) found that corporate managers of chain supermarkets and owners of grocery stores in the U.S considered supplier or manufacturer control/contracts as one of the key barriers (4th in importance) to the implementation of healthy food retail strategies. Gravlee et al. (46) indicated that the success of healthy food strategies largely depended on supplier ability to maintain healthy product stocks. Pinard et al. (47) found that producer and supplier agreements put constraints on food retailers’ decision-making to offer healthier products. Literature indicated that the percentage of healthy items sold, and net profit were also key factors in retailer decision-making. For example, a qualitative study indicated that increasing sales was a key factor for supermarket and grocery store managers and owners when deciding whether to implement healthy retail strategies (46). Another study indicated that commercial viability in terms of increasing percentage of healthy items sold and profits were the two main priorities in retailer decision-making (48).

The attribute “ease of implementation” was not found to be statistically significant (*p* = 0.311) in the total sample. However, qualitative study have emphasised that owners and managers considered ‘ease of implementation’ as an important factor in the adoption of healthy strategies in supermarkets and grocery stores (45). Our study highlights a difficulty with trading off attributes. Results suggest that the ‘ease of implementation’ attribute may not be viewed as important compared to other attributes such as customer satisfaction, profit, retailer satisfaction, and the percentage of healthy items sold. Additionally, given the diversity of the retailers’ position (owners, managers) and retail settings (chain supermarket, independent grocery stores), the significance of “ease of implementation” might vary across different retailers. The non-significant results may be influenced by the small sample size of grocery store respondents.

The magnitude of retailers’ WTP for various attribute levels varied from A$650 per year for a strategy that increased the net profit by 3% to A$32,136 per year for a strategy that led to higher customer satisfaction compared to the reference level. Subgroup analysis showed that there were differences in the mWTP estimates between chain supermarket managers and independent grocery store owners/managers with grocery store responders willing to pay smaller amounts for all attributes. There are several potential reasons for these differences. First, larger chain supermarkets have greater financial resources and economies of scale, allowing them to potentially invest more in healthy food retail strategies (49). In contrast, smaller independent grocery stores have limited financial resources and tend to focus on day-to-day operations (50). Therefore, smaller stores may be more constrained when investing in long-term strategies aimed at improving public health. Second, independent grocery stores may face operational limitations, including lack of space, infrastructure, equipment (such as refrigerators and display equipment, promotional materials), as well as limited human resources, making the implementation of healthy food interventions more costly and time-consuming (50).

Our results showed that only 73 observations (3.3%) were counted for the opt-out scenario. This demonstrated that managers and owners of Australian supermarkets and grocery stores were willing to implement healthy food retail strategies in stores as long as certain business outcomes were achieved. This is consistent with the literature that many supermarket retailers have implemented strategies to improve population health (51).

This study has several strengths. To our knowledge, this is the first study that has used a DCE to elicit retailer preferences and estimate their mWTP for various business outcomes of health promoting food retail interventions. Including the cost attribute allowed us to quantify the monetary value of different attributes which can then be incorporated into economic evaluations of various food retail strategies to assess the cost-effectiveness of these interventions from a broad societal perspective. Using an iterative process (including a literature review, discussion among the research team, consultation with experts, and a cross-sectional survey) to inform the selection of attributes and pilot testing of the survey with academics who worked with retailers and also with retailers, strengthened the validity of the survey design and results. The internal validity test suggested that our results are robust after excluding participants who failed the test.

This study also has some limitations. The study has a relatively small sample size (in particular for the subgroup analysis for grocery store respondents due to the difficulty in recruiting this specific target population). This may have contributed to the insignificant estimates in our analyses and may limit the generalisability of the subgroup analysis. Like all DCEs, this study presented the respondents with hypothetical scenarios, and therefore there might be some concerns about the external validity of our results. This could potentially lead to overestimation of the results, as respondents were not faced with the real-world consequences of their stated choices (52). However, to improve the external validity bias, we included an opt-out option to reflect a real-world scenario. Previous studies have not used a DCE or any other preference elicitation technique to assess how retailers quantify and trade-off business outcomes when deciding whether to implement healthy food retail strategies in supermarkets and grocery stores. This limits the comparison of study results with findings from similar real-world scenarios. Future studies should consider using an integrated approach that combines both stated preference and revealed preference methods to improve the accuracy and reduce bias of results (29). However, using integrated methods would necessarily entail substantially higher cost and time.

Since the opinions of chain store managers would be likely influenced by the corporate strategy and culture, future studies need to assess the opinions of those at different levels of management and decision-making such as store managers, communication managers, social responsibility managers, managing directors, Chief Executive Officers (CEOs), etc. Additionally, this study does not compare the preferences of current and previous owners/managers in their decision making regarding the importance of various business outcomes. The decision-making process of owners or managers is influenced by a variety of external and internal factors that can evolve over-time. Previous owners or managers may have made decisions based on market trends (e.g., economic shocks, technological innovations), consumer behaviour, and corporate strategies prevalent at the time, which may differ from those faced by current owners and managers (29). Another limitation of this study is that the MNL did not take into consideration preference heterogeneity among respondents. The model assumed that everyone in the population has the same preferences and therefore the same utility. However, people’s preferences can vary due to factors such as personal characteristics, age, gender, income, and education. Therefore, future research should consider using alternative methods such as mixed logit or random parameters logit to account for preference differences among respondents (41).

Finally, this study was limited to Australian supermarkets and grocery stores, which may limit the generalizability of the results to other retail settings (i.e., school canteen, restaurants, etc.) and other countries. Further research is needed to evaluate the applicability of the results in different retail settings, locations, and economic circumstances. For instance, the business impact of healthy food retail initiatives implemented in restaurants or school cafeterias may vary from those in supermarkets and grocery stores. Additionally, the nature of the Australian food retail market may differ from other countries, potentially leading to different priorities for retailers. In comparison to other countries, Australia’s level of food market concentration is notably higher (44). This concentration leads to limited competition, and smaller suppliers often experience pressure from these dominant players to meet strict price and supply chain requirements. By contrast, the U.S. and European grocery markets are more fragmented (44). This may affect the transferability of the results into other countries; however, this study provides a foundation for adaptation to other retail settings and country contexts.

The knowledge generated from this study has a range of potential applications. Healthy food retail strategies align with the Australian National Obesity Strategy (53) and the Australian National Preventive Health Strategy (54). The results could be incorporated into a cost–benefit analysis of selected healthy food retail strategies to inform government decision-makers on the value for money of these strategies from a broad societal perspective. The results could assist researchers identify interventions that are likely to engage food retailers to improve the availability, affordability, and acceptability of healthier food products.

## 5 Conclusion

Retailer business outcomes are key variables that may determine the success of healthy food retail strategies. This research provides novel evidence on how retailers quantify and trade-off business outcomes that are important to food retailers in their decision-making to implement health promoting strategies. The knowledge generated from this study will have wide-ranging applicability. The results could inform a range of stakeholders including government, public health specialists, and food retailers to develop and adopt strategies to increase the healthiness of food retail environments that appeal most to food retailers in Australia. Quantifying retailer preferences in terms of their mWTP for various business outcomes and incorporating them into the economic evaluation of various healthy food retail strategies will help generate evidence on which strategies represent good value-for-money from a broad societal perspective and therefore enhance implementation and sustainment.

## Data Availability

The raw data supporting the conclusions of this article will be made available by the authors, without undue reservation.
