# Supplementary material for: How supermarket retailers value business outcomes of healthy food retail strategies: a discrete choice experiment
Source: Front Public Health. 2024 Nov 8;12:1450080. doi: 10.3389/fpubh.2024.1450080 (PMC11582052; doi:10.3389/fpubh.2024.1450080)
Supplement: Supplementary file 1 [file Data_Sheet_1.docx]

**TITLE: How supermarket retailers value business outcomes of healthy food retail strategies: a discrete choice experiment**

**Supplementary file 1: The ESTIMATE checklists for DCEs**

| ESTIMATE | Recommendation | Page |
| --- | --- | --- |
| Estimates | Describe the choice of parameter estimates resulting from the model appropriately and completely, including.   - Whether each variable corresponds to an effects-coded level, a dummy-coded level, or a continuous change in level. - Whether each variable corresponded to the main effect or interaction effect. - Whether continuous variables are linear or have alternative functional form. | Pages 9-10; Table 1 |
| Stochastic | Describe the stochastic properties of the analysis, including.   - The statistical distributions of parameter estimates. - The distribution of parameter estimates across the sample (preferences heterogeneity). - The variance of the estimation function, including systematic differences in variance across observations (scale heterogeneity). | Pages 9-10 |
| Trade-offs | Describe the trade-off that can be inferred from the model, including.   - The magnitude and direction of the attribute-level-coefficients. - The relative importance of each attribute over the range of levels included in the experiment. - The rate at which respondents are willing to trade off among the attributes (marginal rate of substitution). | Pages 10-15 |
| Interpretation | Provide interpretation of the results taking into account the properties of the statistical model, including   - Conclusion that can be drown from the results. - Applicability of the sample, including subgroups or segments, to the population of interests. - Limitations of the results. | Pages 19-19  Pages 4-5  Pages 17-18 |
| Method | Describe the reasons for selecting the statistical analysis method used in the analysis including.   - Why the method is appropriate for analysing the data generated by the experiment. - Why the method is appropriate for addressing the underlying research questions. - Why the method is selected over alternative methods | Pages 9-10 |
| Assumptions | Describe the assumptions of the model and the implications of the assumptions for interpreting the results, including.   - Assumptions about the error of distribution. - Assumptions about the independent observations. - Assumptions about the functional form of the value function. | NA |
| Transparent | Describe the study in sufficiently transparent way to warrant replication, including descriptions.   - The data setup, including handling missing data. - The estimation function, including the value function and the statistical analysis method. - The software used for estimation. | Pages 9-10 |
| Evaluation | Provide an evaluation of the appropriateness of the statistical analysis method to answering the research question, including.   - The goodness of fit of the model. - Sensitivity analysis of the model specification. - Consistency of the results estimated using different methods. | Table 3, Table 4  Table 4  NA |

NA = not applicable

**Supplementary file 2: Final D-efficient design**


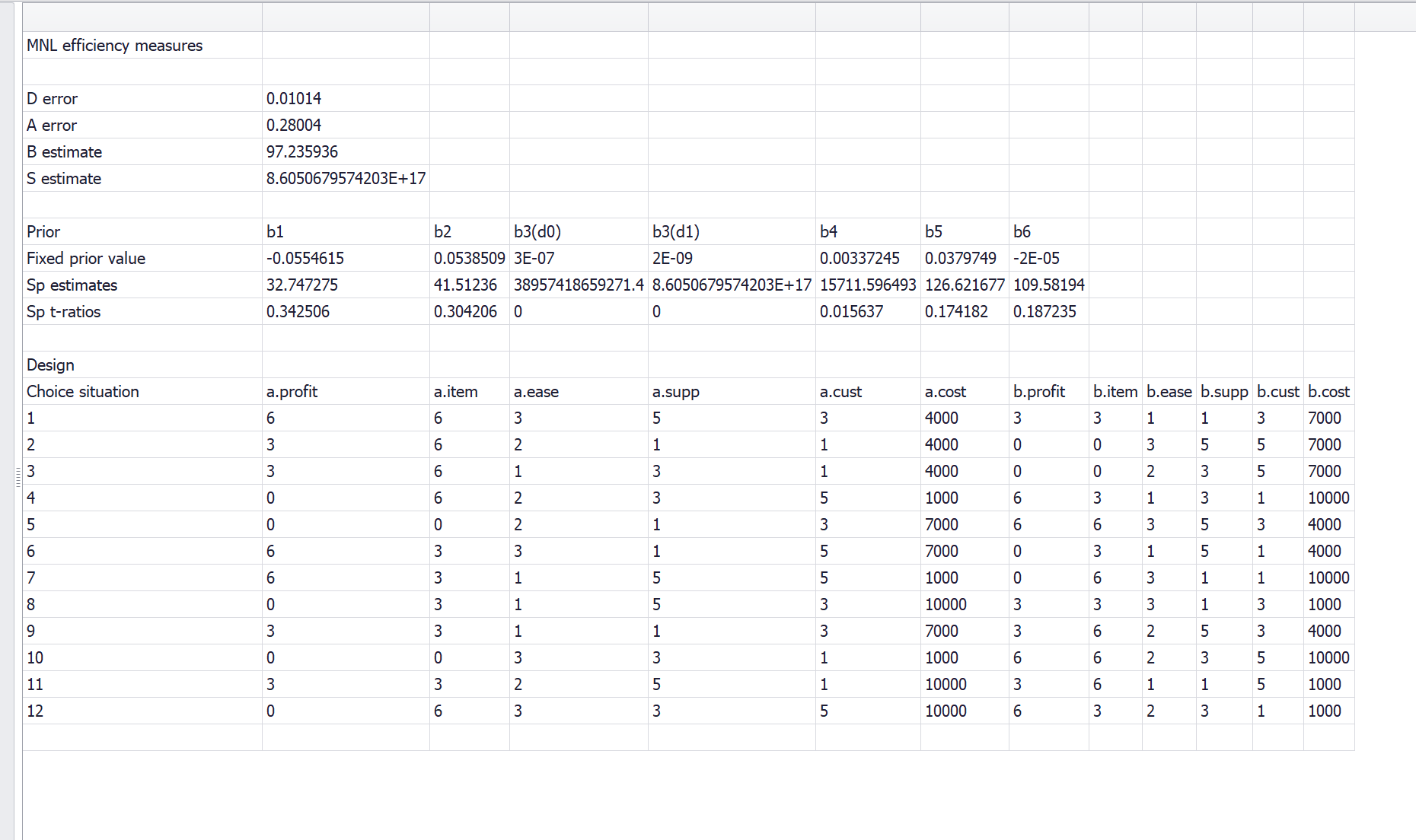


MNL: multinominal logit model; sup: supplier; cust: customer

**Supplementary file 3: The survey**

**Retailers’ preferences when considering using healthy food strategies in Australian supermarkets and grocery stores.**

The food eaten by Australians has a big influence on their health. Supermarkets and grocery stores are the main source of food for many people. Strategies (business practices or operations) promoting healthier food within supermarkets may encourage purchasing and consumption of healthier foods. These may include pricing strategies such as discounts on healthy food, placement or positioning of healthy food at prime locations such as near check outs, increased availability of healthy alternatives, and promotion of healthy products within stores such as through advertising, nutritional labelling, and signage. The success of the strategies depends on how much they align with the core business of the store. As a manager or owner within the food retail sector, you will have a good understanding of factors that influence the use of such strategies to promote healthier products in your store. Finding out more about the impact of retail strategies targeting healthier foods on business outcomes (key performance indicators) will help identify strategies that are both good for health and the retail business. **The aim of this project is to quantify the value of various business outcomes that are considered when implementing healthy food strategies from the perspective of Australian supermarket and grocery stores.**

If you would like to be involved in this project, please review the Plain Language Statement and Consent Form below and then proceed with the survey. If you have any questions or require further information, please contact ……….

- Attached PDF file of Plain Language Statement

**I have read the plain language statement and I agree to participate (Clickable)**

1. Yes
2. No

**Part one: Screening Questions:**

**Are you currently working in or previously worked in a supermarket or grocery store in Australia and had some decision-making experience related to the adoption of strategies promoting healthier products?**

1. Yes
2. No

*[If response is NO then NOT eligible to participate.] Thank you for considering taking part in this survey. Based on the responses you provided you are not eligible to participate.*

**Which of the following positions have you held or currently hold within grocery stores or supermarkets? Select all that apply.**

1. Owner
2. Manager
3. Other, please specify…………

**Part 2: Discrete Choice Experiment Scenario**

The following section consists of 13 choice tasks. Each choice task will present you with two hypothetical strategies that have varied impacts on 6 different business outcomes that have been identified as important when considering implementing strategies that promote healthier products in your store. We would like you to think about each choice task as if you were making a decision about which strategy you would use in your store. Then we will ask you to tell us which strategy 1 or 2 you most prefer. You can state that you prefer to remain in the current situation (not to implement either strategy) by choosing option 3 “Neither option”.

**This is a practice task. In this task strategy 2 has more favourable characteristics for each option.”**

**Table 1: Example of the choice task 1.**

| **Characteristics (attributes)** | **Strategy A** | **Strategy B** | **Option C** |
| --- | --- | --- | --- |
| The change in the store’s net profit. | 3% increase in store’s net profit | 6% increase in store’s net profit | **None of them** |
| The change in percentage (%) of healthy items sold. | 3% increase of healthy items sold. | 6% increase of healthy items sold. |  |
| Ease of implementation for employee. | ★✰✰✰✰  (Difficult) | ★★★✰✰  (Neither easy nor difficult) |  |
| Satisfaction of food suppliers and producers with the strategy promoting healthier products. | ★★★✰✰  (Neutral) | ★★★★★  (Very satisfied) |  |
| Customer satisfaction with store overall as a result with the strategy. | ★✰✰✰✰  (Very unsatisfied) | ★★★★★  (Very satisfied) |  |
| Start up and running costs associated with putting in place and maintaining strategies to promote healthier products | A$4,000 | A$1,000 |  |
| **Tick the ONE you prefer the most.** | ⃝ | ⃝ | ⃝ |

**Choice task 1:**

| **Characteristics (attributes)** | **Strategy A** | **Strategy B** | **Option C** |
| --- | --- | --- | --- |
| The change in the store’s net profit. | 6% increase in store’s net profit | 3% increase in store’s net profit | **None of them** |
| The change in percentage (%) of healthy items sold. | 6% increase of healthy items sold. | 3% increase of healthy items sold. |  |
| Ease of implementation for employee. | ★★★✰✰  (Neither easy nor difficult) | ★★★★★  (Easy) |  |
| Satisfaction of food suppliers and producers with the strategy promoting healthier products. | ★★★★★  (Very satisfied) | ★✰✰✰✰  (Very unsatisfied) |  |
| Customer satisfaction with store overall as a result with the strategy. | ★★★✰✰  (Neutral) | ★★★✰✰  (Neutral) |  |
| Start up and running costs associated with putting in place and maintaining strategies to promote healthier products | A$4,000 | A$7,000 |  |
| **Tick the ONE you prefer the most.** | ⃝ | ⃝ | ⃝ |

**Choice task 2:**

| **Characteristics (attributes)** | **Strategy A** | **Strategy B** | **Option C** |
| --- | --- | --- | --- |
| The change in the store’s net profit. | 3% increase in store’s net profit | 0% increase in store’s net profit | **None of them** |
| The change in percentage (%) of healthy items sold. | 6% increase of healthy items sold. | 0% increase of healthy items sold. |  |
| Ease of implementation for employee. | ★✰✰✰✰  (Difficult) | ★★★✰✰  (Neither easy nor difficult) |  |
| Satisfaction of food suppliers and producers with the strategy promoting healthier products. | ★✰✰✰✰  (Very unsatisfied) | ★★★★★  (Very satisfied) |  |
| Customer satisfaction with store overall as a result with the strategy. | ★✰✰✰✰  (Very unsatisfied) | ★★★★★  (Very satisfied) |  |
| Start up and running costs associated with putting in place and maintaining strategies to promote healthier products | A$4,000 | A$7,000 |  |
| **Tick the ONE you prefer the most.** | ⃝ | ⃝ | ⃝ |

**Choice task 3:**

| **Characteristics (attributes)** | **Strategy A** | **Strategy B** | **Option C** |
| --- | --- | --- | --- |
| The change in the store’s net profit. | 3% increase in store’s net profit | 0% increase in store’s net profit | **None of them** |
| The change in percentage (%) of healthy items sold. | 6% increase of healthy items sold. | 0% increase of healthy items sold. |  |
| Ease of implementation for employee. | ★★★★★  (Easy) | ★✰✰✰✰  (Difficult) |  |
| Satisfaction of food suppliers and producers with the strategy promoting healthier products. | ★★★✰✰  (Neutral) | ★★★✰✰  (Neutral) |  |
| Customer satisfaction with store overall as a result with the strategy. | ★✰✰✰✰  (Very unsatisfied) | ★★★★★  (Very satisfied) |  |
| Start up and running costs associated with putting in place and maintaining strategies to promote healthier products | A$4,000 | A$7,000 |  |
| **Tick the ONE you prefer the most.** | ⃝ | ⃝ | ⃝ |

**Choice task 4:**

| **Characteristics (attributes)** | **Strategy A** | **Strategy B** | **Option C** |
| --- | --- | --- | --- |
| The change in the store’s net profit. | 0% increase in store’s net profit | 6% increase in store’s net profit | **None of them** |
| The change in percentage (%) of healthy items sold. | 6% increase of healthy items sold. | 3% increase of healthy items sold. |  |
| Ease of implementation for employee. | ★✰✰✰✰  (Difficult) | ★★★★★  (Easy) |  |
| Satisfaction of food suppliers and producers with the strategy promoting healthier products. | ★★★✰✰  (Neutral) | ★★★✰✰  (Neutral) |  |
| Customer satisfaction with store overall as a result with the strategy. | ★★★★★  (Very satisfied) | ★✰✰✰✰  (Very unsatisfied) |  |
| Start up and running costs associated with putting in place and maintaining strategies to promote healthier products | A$1,000 | A$10,000 |  |
| **Tick the ONE you prefer the most.** | ⃝ | ⃝ | ⃝ |

**Choice task 5:**

| **Characteristics (attributes)** | **Strategy A** | **Strategy B** | **Option C** |
| --- | --- | --- | --- |
| The change in the store’s net profit. | 0% increase in store’s net profit | 6% increase in store’s net profit | **None of them** |
| The change in percentage (%) of healthy items sold. | 0% increase of healthy items sold. | 6% increase of healthy items sold. |  |
| Ease of implementation for employee. | ★✰✰✰✰  (Difficult) | ★★★✰✰  (Neither easy nor difficult) |  |
| Satisfaction of food suppliers and producers with the strategy promoting healthier products. | ★✰✰✰✰  (Very unsatisfied) | ★★★★★  (Very satisfied) |  |
| Customer satisfaction with store overall as a result with the strategy. | ★★★✰✰  (Neutral) | ★★★✰✰  (Neutral) |  |
| Start up and running costs associated with putting in place and maintaining strategies to promote healthier products | A$7,000 | A$4,000 |  |
| **Tick the ONE you prefer the most.** | ⃝ | ⃝ | ⃝ |

**Choice task 6:**

| **Characteristics (attributes)** | **Strategy A** | **Strategy B** | **Option C** |
| --- | --- | --- | --- |
| The change in the store’s net profit. | 6% increase in store’s net profit | 0% increase in store’s net profit | **None of them** |
| The change in percentage (%) of healthy items sold. | 3% increase of healthy items sold. | 3% increase of healthy items sold. |  |
| Ease of implementation for employee. | ★★★✰✰  (Neither easy nor difficult) | ★★★★★  (Easy) |  |
| Satisfaction of food suppliers and producers with the strategy promoting healthier products. | ★✰✰✰✰  (Very unsatisfied) | ★★★★★  (Very satisfied) |  |
| Customer satisfaction with store overall as a result with the strategy. | ★★★★★  (Very satisfied) | ★✰✰✰✰  (Very unsatisfied) |  |
| Start up and running costs associated with putting in place and maintaining strategies to promote healthier products | A$7,000 | A$4,000 |  |
| **Tick the ONE you prefer the most.** | ⃝ | ⃝ | ⃝ |

**Choice task 7:**

| **Characteristics (attributes)** | **Strategy A** | **Strategy B** | **Option C** |
| --- | --- | --- | --- |
| The change in the store’s net profit. | 6% increase in store’s net profit | 0% increase in store’s net profit | **None of them** |
| The change in percentage (%) of healthy items sold. | 3% increase of healthy items sold. | 6% increase of healthy items sold. |  |
| Ease of implementation for employee. | ★★★★★  (Easy) | ★★★✰✰  (Neither easy nor difficult) |  |
| Satisfaction of food suppliers and producers with the strategy promoting healthier products. | ★★★★★  (Very satisfied) | ★✰✰✰✰  (Very unsatisfied) |  |
| Customer satisfaction with store overall as a result with the strategy. | ★★★★★  (Very satisfied) | ★✰✰✰✰  (Very unsatisfied) |  |
| Start up and running costs associated with putting in place and maintaining strategies to promote healthier products | A$1,000 | A$10,000 |  |
| **Tick the ONE you prefer the most.** | ⃝ | ⃝ | ⃝ |

**Choice task 8:**

| **Characteristics (attributes)** | **Strategy A** | **Strategy B** | **Option C** |
| --- | --- | --- | --- |
| The change in the store’s net profit. | 0% increase in store’s net profit | 3% increase in store’s net profit | **None of them** |
| The change in percentage (%) of healthy items sold. | 3% increase of healthy items sold. | 3% increase of healthy items sold. |  |
| Ease of implementation for employee. | ★★★★★  (Easy) | ★★★✰✰  (Neither easy nor difficult) |  |
| Satisfaction of food suppliers and producers with the strategy promoting healthier products. | ★★★★★  (Very satisfied) | ★✰✰✰✰  (Very unsatisfied) |  |
| Customer satisfaction with store overall as a result with the strategy. | ★★★✰✰  (Neutral) | ★★★✰✰  (Neutral) |  |
| Start up and running costs associated with putting in place and maintaining strategies to promote healthier products | A$10,000 | A$1,000 |  |
| **Tick the ONE you prefer the most.** | ⃝ | ⃝ | ⃝ |

**Choice task 9:**

| **Characteristics (attributes)** | **Strategy A** | **Strategy B** | **Option C** |
| --- | --- | --- | --- |
| The change in the store’s net profit. | 3% increase in store’s net profit | 3% increase in store’s net profit | **None of them** |
| The change in percentage (%) of healthy items sold. | 3% increase of healthy items sold. | 6% increase of healthy items sold. |  |
| Ease of implementation for employee. | ★★★★★  (Easy) | ★✰✰✰✰  (Difficult) |  |
| Satisfaction of food suppliers and producers with the strategy promoting healthier products. | ★✰✰✰✰  (Very unsatisfied) | ★★★★★  (Very satisfied) |  |
| Customer satisfaction with store overall as a result with the strategy. | ★★★✰✰  (Neutral) | ★★★✰✰  (Neutral) |  |
| Start up and running costs associated with putting in place and maintaining strategies to promote healthier products | A$7,000 | A$4,000 |  |
| **Tick the ONE you prefer the most.** | ⃝ | ⃝ | ⃝ |

**Choice task 10:**

| **Characteristics (attributes)** | **Strategy A** | **Strategy B** | **Option C** |
| --- | --- | --- | --- |
| The change in the store’s net profit. | 0% increase in store’s net profit | 6% increase in store’s net profit | **None of them** |
| The change in percentage (%) of healthy items sold. | 0% increase of healthy items sold. | 6% increase of healthy items sold. |  |
| Ease of implementation for employee. | ★★★✰✰  (Neither easy nor difficult) | ★✰✰✰✰  (Difficult) |  |
| Satisfaction of food suppliers and producers with the strategy promoting healthier products. | ★★★✰✰  (Neutral) | ★★★✰✰  (Neutral) |  |
| Customer satisfaction with store overall as a result with the strategy. | ★✰✰✰✰  (Very unsatisfied) | ★★★★★  (Very satisfied) |  |
| Start up and running costs associated with putting in place and maintaining strategies to promote healthier products | A$1,000 | A$10,000 |  |
| **Tick the ONE you prefer the most.** | ⃝ | ⃝ | ⃝ |

**Choice task 11:**

| **Characteristics (attributes)** | **Strategy A** | **Strategy B** | **Option C** |
| --- | --- | --- | --- |
| The change in the store’s net profit. | 3% increase in store’s net profit | 3% increase in store’s net profit | **None of them** |
| The change in percentage (%) of healthy items sold. | 3% increase of healthy items sold. | 6% increase of healthy items sold. |  |
| Ease of implementation for employee. | ★✰✰✰✰  (Difficult) | ★★★★★  (Easy) |  |
| Satisfaction of food suppliers and producers with the strategy promoting healthier products. | ★★★★★  (Very satisfied) | ★✰✰✰✰  (Very unsatisfied) |  |
| Customer satisfaction with store overall as a result with the strategy. | ★✰✰✰✰  (Very unsatisfied) | ★★★★★  (Very satisfied) |  |
| Start up and running costs associated with putting in place and maintaining strategies to promote healthier products | A$10,000 | A$1,000 |  |
| **Tick the ONE you prefer the most.** | ⃝ | ⃝ | ⃝ |

**Choice task 12:**

| **Characteristics (attributes)** | **Strategy A** | **Strategy B** | **Option C** |
| --- | --- | --- | --- |
| The change in the store’s net profit. | 0% increase in store’s net profit | 6% increase in store’s net profit | **None of them** |
| The change in percentage (%) of healthy items sold. | 6% increase of healthy items sold. | 3% increase of healthy items sold. |  |
| Ease of implementation for employee. | ★★★✰✰  (Neither easy nor difficult) | ★✰✰✰✰  (Difficult) |  |
| Satisfaction of food suppliers and producers with the strategy promoting healthier products. | ★★★✰✰  (Neutral) | ★★★✰✰  (Neutral) |  |
| Customer satisfaction with store overall as a result with the strategy. | ★★★★★  (Very satisfied) | ★✰✰✰✰  (Very unsatisfied) |  |
| Start up and running costs associated with putting in place and maintaining strategies to promote healthier products | A$10,000 | A$1,000 |  |
| **Tick the ONE you prefer the most.** | ⃝ | ⃝ | ⃝ |

**Choice task 13: (repeated choice to test validity)**

| **Characteristics (attributes)** | **Strategy A** | **Strategy B** | **Option C** |
| --- | --- | --- | --- |
| The change in the store’s net profit. | 0% increase in store’s net profit | 6% increase in store’s net profit | **None of them** |
| The change in percentage (%) of healthy items sold. | 0% increase of healthy items sold. | 6% increase of healthy items sold. |  |
| Ease of implementation for employee. | ★✰✰✰✰  (Difficult) | ★★★✰✰  (Neither easy nor difficult) |  |
| Satisfaction of food suppliers and producers with the strategy promoting healthier products. | ★✰✰✰✰  (Very unsatisfied) | ★★★★★  (Very satisfied) |  |
| Customer satisfaction with store overall as a result with the strategy. | ★★★✰✰  (Neutral) | ★★★✰✰  (Neutral) |  |
| Start up and running costs associated with putting in place and maintaining strategies to promote healthier products | A$7,000 | A$4,000 |  |
| **Tick the ONE you prefer the most.** | ⃝ | ⃝ | ⃝ |

**Part 3: Respondent characteristics**

**In which State or Territory are/were the store/s in which you have worked in located? Select all that apply.**

1. Australian Capital Territory
2. New South Wales
3. Victoria
4. Northern Territory
5. Queensland
6. South Australia
7. Tasmania
8. Western Australia

**What type of stores have you worked in/owned? Select all that apply**

1. Chain supermarket
2. Independent grocery store including IGA, Foodworks stores, and family grocery stores.
3. Other please specify…………..

**How long have you worked in or owned supermarkets or grocery stores?**

1. Less than 1 year
2. Between and 1 5 years
3. Between 6 and 10 years
4. More than 10 years

**Are there any other comments you would like to comment about the survey?**

|  |
| --- |

**Your participation will help enhance the health of Australians. When you click SUBMIT, the survey will be complete, and you are consenting to the completed confidential survey being submitted and used in this research.**

**We thank you for your time spent taking this survey.**

**Your response has been recorded**.

**Supplementary file 4: Results of the linear conditional logit model (CL) for chain supermarkets**

| Attribute (business outcomes) | Coefficient | SE | [95% CI] | P value |
| --- | --- | --- | --- | --- |
| Net profit | 0.047 | 0.022 | [0.003; 0.090] | 0.090 |
| (%) of healthy items sold | 0.167 | 0.032 | [0.105; 0.230] | 0.229 |
| Ease of implementation | 0.126 | 0.101 | [-0.073; 0.326] | 0.215 |
| Supplier/producer satisfaction | 0.417 | 0.084 | [0.252; 0.580] | < 0.001 |
| Customer satisfaction | 0.652 | 0.097 | [0.462; 0.842] | < 0.001 |
| Implementation costs | - 0.0000227 | 0.000021 | [- 0.0000637; 0.0000184] | 0.279 |
| Number of observations: 1,509  Wald chi2 (6): 81.90  Prob > chi2: 0.0000  Log pseudo likelihood: - 384.101 | | | | |

SE = standard error; CI= confidence interval

**Supplementary file 5: Results of the linear conditional logit model (CL) for independent grocery stores**

| Attribute (business outcomes) | Coefficient | SE | [95% CI] | P value |
| --- | --- | --- | --- | --- |
| Net profit | 0.059 | 0.035 | [-0.009; 0.127] | 0.091 |
| (%) of healthy items sold | 0.066 | 0.041 | [-0.015; 0.146] | 0.109 |
| Ease of implementation | -0.020 | 0.085 | [-0.185; 0.146] | 0.817 |
| Supplier/producer satisfaction | 0.290 | 0.099 | [0.096; 0.484] | 0.003 |
| Customer satisfaction | 0.347 | 0.147 | [0.059; 0.634] | 0.018 |
| Implementation costs | -0.0000409 | 0.0000305 | [-0.0001; 0.0000189] | 0.180 |
| Number of observations: 681  Wald chi2 (6): 22.81  Prob > chi2: 0.0009  Log pseudo likelihood: - 212.415 | | | | |

SE = standard error; CI= confidence interval

**Supplementary file 6: Results of the linear conditional model (all respondents) after excluding participants who failed the validity test.**

| Attribute (business outcomes) | Coefficient | SE | [95% CI] | P value |
| --- | --- | --- | --- | --- |
| Net profit | 0.063 | 0.020 | [0.023; 0.102] | 0.002 |
| (%) of healthy items sold | 0.151 | 0.027 | [0.097; 0.204] | < 0.001 |
| Ease of implementation | 0.079 | 0.075 | [-0.067; 0.225] | 0.290 |
| Supplier/producer satisfaction | 0.386 | 0.068 | [0.253; 0.518] | < 0.001 |
| Customer satisfaction | 0.435 | 0.081 | [0.276; 0.594] | < 0.001 |
| Implementation costs | - 0.0000432 | 0.0000163 | [- 0.0000753; 0.0000112] | 0.008 |
| Number of observations: 1,797  Wald chi2 (6): 85.87  Prob > chi2: 0.0000  Log pseudo likelihood: - 482.62093 | | | | |

SE = standard error; CI= confidence interval

**Supplementary file 7: Results of the Relative importance of business outcomes after excluding participants who failed the validity test**

| Attribute (business outcome) | Attribute level | All respondents (n=50) | | |
| --- | --- | --- | --- | --- |
|  |  | Coefficient (SE) | [95% CI] | P value |
| Customer satisfaction | Very unsatisfied (reference) | | | |
|  | Neutral | 0.800 (0.385) | [0.046; 1.554] | 0.038 |
|  | Very satisfied | 0.811 (0.256) | [0.308; 1.313] | 0.002 |
| Percentage (%) of healthy items sold | 0% (reference) | | | |
|  | 3% increase | 0.193 (0.335) | [-0.463; 0.849] | 0.563 |
|  | 6% increase | 0.768 (0.349) | [0.083; 1.452] | 0.028 |
| Supplier/producer satisfaction | Very unsatisfied (reference) | | | |
|  | Neutral | 0.567 (0.360) | [-0.139; 1.273] | 0.116 |
|  | Very satisfied | 0.750 (0.261) | [0.240; 1.126] | 0.004 |
| Net profit | 0% (reference) | | | |
|  | 3% increase | 0.215 (0.244) | [-0.262; 0.693] | 0.377 |
|  | 6% increase | 0.464 (0.171) | [0.130; 0.799] | 0.007 |
| Ease of implementation | Difficult (reference) | | | |
|  | Neither easy nor difficult | 0.065 (0.141) | [-0.212; 0.342] | 0.645 |
|  | Easy | 0.123 (0.157) | [-0.186; 0.432] | 0.435 |
| Implementation costs | A$1,000 (reference) | | | |
|  | A$4,000 | -0.198 (0.399) | [-0.979; 0.584] | 0.620 |
|  | A$7,000 | -0.299 (0.363) | [-1.011; 0.413] | 0.411 |
|  | A$10,000 | -0.422 (0.206) | [-0.827; -0.018] | 0.040 |
|  | | Number of observations: 1,797  Wald chi2 (6): 104.09  Prob > chi2: p<0.0001  Log pseudo likelihood: - 481.65408 | | |

SE = standard error; CI= confidence interval

**Supplementary file 8: Marginal willingness to pay for attribute levels across grocery store respondents.**

| Attribute (business outcome) | Attribute level | Coefficient (SE) | WTP (A$, 95% CI) |
| --- | --- | --- | --- |
| Customer satisfaction | Very unsatisfied (reference) | | |
|  | Neutral | 0.404 (0.522) | 9,330 [-14,298; 32,958] |
|  | Very satisfied | 0.530 (0.306) | 12,240 [-1,611; 26,091] |
| (%) of healthy items sold | 0% (reference) | | |
|  | 3% increase | -0.253 (0.523) | -5,843 [-29,518; 17,832] |
|  | 6% increase | 0.247 (0.353) | 5,704 [-10,274; 21,682] |
| Supplier/producer satisfaction | Very unsatisfied (reference) | | |
|  | Neutral | 0.099 (0.476) | 2,302 [-19,244; 23,848] |
|  | Very satisfied | 0.396 (0.241) | 9,145 [-1,764; 20,054] |
| Net profit | 0% (reference) | | |
|  | 3% increase | -0.106 (0.282) | -2,448 [-15,213; 10,317] |
|  | 6% increase | 0.537 (0.288) | 12,402 [-634; 25,438] |
| Ease of implementation | Difficult (reference) | | |
|  | Neither easy nor difficult | 0.056 (0.216) | 1,293 [-9,775; 11,069] |
|  | Easy | 0.163 (0.219) | 3,764 [-6,150; 13,678] |
| Implementation costs (Per year) | Linear | -0.0000433 | - |

SE: standard error; CI: confidence interval; WTP: willingness to pay

Statistical significance is indicated at the 1% (***), 5% (**)

**Supplementary file 9: Marginal willingness to pay for attribute levels across chain supermarket respondents.**

| Attribute (business outcome) | Attribute level | Coefficient (SE) | WTP [A$, 95% CI] |
| --- | --- | --- | --- |
| Customer satisfaction | Very unsatisfied (reference) | | |
|  | Neutral | 1.187 (0.405) *** | 50,726 [16,802; 84,650] |
|  | Very satisfied | 1.339 (0.232) *** | 57,222 [37,789; 76,655] |
| (%) of healthy items sold | 0% (reference) | | |
|  | 3% increase | 0.580 (0.307) | 24,786 [-929; 50,501] |
|  | 6% increase | 1.035 (0.236) *** | 44,231 [24,464; 63,998) |
| Supplier/producer satisfaction | Very unsatisfied (reference) | | |
|  | Neutral | 0.652 (0.381) | 27,863 [-4,050; 59,776] |
|  | Very satisfied | 0.821 (0.215) *** | 35,085 [17,077; 53,093] |
| Net profit | 0% (reference) | | |
|  | 3% increase | 0.111 (0.302) | 4,744 [-20,552; 30,040] |
|  | 6% increase | 0.250 (0.183) | 10,684 [-4,645; 26,013] |
| Ease of implementation | Difficult (reference) | | |
|  | Neither easy nor difficult | 0.211 (0.184) | 9.018 [-6,393; 24,429] |
|  | Easy | 0.254 (0.219) | 10,855 [-7,489; 29,199] |
| Implementation costs (Per year) | Linear | -0.0000234 | - |

SE: standard error; CI: confidence interval; WTP: willingness to pay

Statistical significance is indicated at the 1% (***), 5% (**)
